# Thymocyte development in the absence of matrix metalloproteinase-9/gelatinase B

**DOI:** 10.1038/srep29852

**Published:** 2016-07-19

**Authors:** Natalia V. Gounko, Erik Martens, Ghislain Opdenakker, Vasily Rybakin

**Affiliations:** 1 Laboratory of Immunobiology, REGA Institute, Department of Microbiology and Immunology, KU Leuven, Minderbroedersstraat 10, Leuven 3000, Belgium; 2 Electron Microscopy Platform, Center for the Biology of Disease VIB and Center for Human Genetics KU Leuven, Herestraat 49, 3000 Leuven, Belgium

## Abstract

Matrix metalloproteinases (MMP) play critical roles in a variety of immune reactions by facilitating cell migration, and affect cell communication by processing both cytokines and cell surface receptors. Based on published data indicating that MMP-9 is upregulated upon T cell activation and also in the thymus upon the induction of negative selection, we investigated the contribution of MMP-9 into mouse T cell development and differentiation in the thymus. Our data suggest that MMP-9 deficiency does not result in major abnormalities in the development of any conventionally selected or agonist selected subsets and does not interfere with thymocyte apoptosis and clearance, and that MMP-9 expression is not induced in immature T cells at any stage of their thymic development.

Gelatinase B/matrix metalloproteinase-9 (MMP-9) is a calcium-dependent, zinc-containing endopeptidase involved into the remodeling of extracellular matrix in a wide variety of biological phenomena, and an increasing body of evidence suggests that it plays an important role in the regulation of multiple immune processes^1^,^2^. It has been well documented that processing of extracellular matrix by MMP-9 is crucial for movement through and invasion into tissues by multiple cell types, including cancer cells, neutrophils, macrophages, dendritic and T cells^3^,^4^,^5^,^6^,^7^,^8^,^9^,^10^. MMP-9-mediated cleavage has also been shown to regulate the activity of a number of cytokines and chemokines^11^,^12^,^13^,^14^,^15^,^16^. MMP-9-deficient mice display elevated levels of autoantibodies to a variety of antigens^17^. A growing body of evidence also indicates that MMP-9 may play a more direct role in the regulation of T cell activation, although the data remain highly fragmented. Cleavage of ICAM-1 by MMP-9 protects cultured breast cancer cells from NK cell-mediated cytotoxicity^18^. It is likely that the same mechanism may also provide immunosuppressive input in both conventional and regulatory T cell-mediated responses since LFA-1 interaction with ICAM-1 is crucial for immune synapse stabilization and T cell activation^19^. Indeed, MMP-9 deficiency results in reduced recruitment of T cells and macrophages and attenuated pathology in experimental glomerulonephritis^5^. Another possible role for MMP-9 arises from the observation that MMP-9 produced by cancer cells cleaves CD25 expressed on the surface of tumor-infiltrating T cells and reduces their proliferation *in vitro*^20^, suggesting that a similar mechanism may downregulate T cell activation *in vivo* by limiting T cell reactivity to IL-2. Chemical inhibition of MMP-9 reduces proliferation of regulatory T cells in the presence of CD3/CD28-coated microbeads *in vitro*, although puzzlingly so does the addition of recombinant MMP-9 in the same study^21^. Finally, both human and mouse T cells and T cell lines have been shown to express and secrete active MMP-9 in response to antigenic stimulation^22^,^23^,^24^, upon chemical stimulation^25^,^26^,^27^,^28^, in response to tumors^29^, and downstream of cell adhesion-related signals^30^,^31^, although the regulation and biological significance of T cell intrinsic production of MMP-9 remain poorly understood. In addition, purity analysis of MMP-9-producing cell populations has not been fully reported in multiple studies.

Direct attempts to evaluate the involvement of MMP-9 into T cell activation by comparing MMP-9-sufficient and -deficient peripheral T cell responses have yielded conflicting data. Using mixed lymphocyte reaction assays, two studies from the same group reported an increased proliferation of MMP-9-deficient T cells upon stimulation with Balb/c dendritic cells, and increased production of IL-12 and IFNγ^32^,^33^. Intriguingly, the authors also observed increased proliferation of wild-type T cells derived from mice stimulated by MMP-9-deficient dendritic cells in mixed lymphocyte reaction, as compared to stimulation with wild-type DC. These data appear to suggest that MMP-9 activity suppresses T cell activation regardless of cell type of origin. In agreement with this conclusion, addition of recombinant MMP-9 to effector T cells stimulated with CD3/CD28-coated beads strongly suppressed their proliferation^21^. However, a later study has revealed a dramatic decrease in the proliferation of MMP-9 knockout T cells as well as in IL-2 and CD25 production in response to stimulation with anti-CD3 antibody^23^. The reasons behind these discrepancies remain to be experimentally addressed.

Odaka and colleagues (2005) reported a transient increase in the expression of MMP-9 in thymic tissue of Balb/c mice 16–24 hours after a single i.p. injection of CD3ε antibody mimicking negative selection^34^. Elevated MMP-9 production coincided with the exposure of cryptic epitopes of collagen IV as revealed by HUIV26 antibody staining, and with increased neovascularization of the thymus^34^. In a separate study, application of the broad spectrum MMP inhibitor GM6001 was shown to delay TCR rearrangement, inhibit the proliferation and progression of thymocytes from CD4^−^CD8^−^ (double negative, DN) to CD4^+^CD8^+^ (double positive, DP) stage in ex vivo thymocyte development assays^35^. Finally, our own data indicated that MMP-9 deficiency combined with *lpr* mutation in the *Fas* gene resulted in thymic hyperplasia^17^. Based on these data, we decided to systematically analyze the requirement for MMP-9 during thymic T cell development, and induction of MMP-9 expression in developing thymocytes in response to stimulation.

## Materials and Methods

### Mice

Experimental protocols were approved by the Ethische Commissie Dierproeven, KU Leuven, animal protocol number B277-2014, laboratory license LA1210251 (Belgium). Animal use was in accordance with institutional permits and KU Leuven ethics policies. MMP-9-deficient mice and MMP-9^−/−^Fas^*Lpr*^ mice were described previously^17^. Here, we used MMP-9^−/−^ after 13 backcrosses into the C57BL/6J strain. MMP-9-sufficient C57BL/6J and Fas^*Lpr*^ control mice were bred and housed in the same vivarium under exactly the same environmental conditions for several years. Photographic images were taken using a Canon EOS3200 camera equipped with a stabilized objective Canon EFS 18–55.

### Antibodies, flow cytometry, and immunohistochemistry

The following antibodies were used for flow cytometry: CD4 (GK1.5), CD5 (53–7.3), CD8β (H35-17.2), CD25 (7D4), CD44 (IM7), F4/80 (BM8), Gr.1 (1A8-Ly6g), TCRβ (H57-597), γδTCR (GL-3), all at 2 μg/ml, and Foxp3 (FJK-16s) at 5 μg/ml, all from either eBioscience or BD Biosciences. Goat polyclonal antibody against MMP-9 antibody used in flow cytometry (2 μg/ml) and immunohistochemistry experiments (2 μg/ml) was from R&D Systems (cat. # AF909). Intracellular staining for MMP-9 was performed using Intracellular Fixation and Permeabilization Buffer Set (eBioscience) and anti-goat secondary antibody labeled with AlexaFluor 647, used at 0.5 μg/ml (ThermoFisher Scientific). Data were acquired on BD LSR Fortessa X-20 flow cytometer and analyzed using FlowJo software, version 7.6.5. Iba1 antibody used for immunohistochemistry was from Wako, used 1:200 (cat. # 019-19741). Immunohistochemical stainings were carried out on a Ventana Ultra staining platform according to manufacturer’s instructions (Roche).

### Thymocyte apoptosis and deletion assays

For *in vivo* induction of thymocyte deletion, three to four 4–5 week old mice per genotype were injected i.p. with 50 μg of anti-CD3ε antibody (clone 145-2C11, purified, sodium azide-free, low endotoxin, BD Biosciences) in 200 μl of sterile PBS, and one sex- and age-matched mouse was injected with 200 μl of sterile PBS. Mice were sacrificed 18 or 72 h post injection. In total, 13–14 CD3ε-injected and 4 PBS-injected mice were used per genotype in four independent injection experiments. For *in vitro* induction of apoptosis, thymi were isolated from 4–5 week old mice, and thymocytes were isolated by pressing the tissue through 70 μm cell strainers (BD). Cells were counted and incubated at 37 °C in 96-well, flat-bottom plates in the presence of CD3/CD28-coated magnetic beads (ThermoFisher Scientific). Cells were harvested after 18 h and analyzed for activation of caspase 3 using CaspGLOW Fluorescein Active Caspase-3 Staining Kit (FITC-VAD-FMK, eBioscience) and for surface expression of Annexin V using Annexin V Apoptosis Detection Kit (eBioscience). Analysis of apoptosis by flow cytometry was performed as described^36^,^37^.

### Electron microscopy

For morphological analysis, thymi were fixed in 2.5% glutaraldehyde, 4% formaldehyde, 0.2% picric acid, in 0.1 M sodium cacodylate buffer (pH 7.4) and stored in the same fixative at 4 °C. Samples were embedded in 3% agarose and sectioned at 150 μm using a vibratome. Sections were postfixed in 1% osmium tetroxide, 1.5% potassium ferrocyanide for 30 min at room temperature, stained with 0.2% tannic acid for 20 min, fixed with 1% osmium tetroxide for 30 min, stained with 1% thiocarbohydrazide for 20 min, and incubated again with 1% osmium tetroxide for 30 min. Samples were contrasted with 0.5% uranyl acetate in 25% methanol overnight at 4 °C and with Walton’s lead acetate for 30 min at 60 °C. After ethanol dehydration, samples were infiltrated and embedded into Agar 100. Blocks were sectioned at 110 nm using a Leica UCT ultra-microtome, collected onto Cu-grids coated with formvar/carbon, and imaged using JEOL JEM1400 transmission electron microscope operated at 80 kV and equipped with an 11-MP Olympus Quemesa camera.

For immunoelectron microscopy, thymi were fixed in 4% formaldehyde in 0.1 M sodium cacodylate buffer (pH 7.4) and stored in the same fixative at 4 °C. Samples were embedded in 3% agarose, sectioned at 150 μm using a vibratome, and small discs were punched out of the sections for freeze-substitution in a Leica AFS2 apparatus. The starting point was at 0 °C in 30% ethanol for 30 min, then lowered to −15 °C to −30 °C in 50% ethanol for 30 min, −30 °C to −35 °C in 75% ethanol for 30 min, and at −35 °C ethanol was replaced with acetone for 1 h. Samples were stained with 0.2% uranyl acetate in acetone for 4 h, washed 3x in acetone, and infiltrated with lowicryl HM20 at −35 °C for 48 h. UV polymerization was initiated for 24 h at −35 °C, then temperature was raised to 20 °C at a rate of 1 degree/h, together with UV polymerization. Blocks were sectioned at 110 nm using a Leica UCT ultra-microtome, and sections were collected on Ni-grids coated with formvar/carbon. Immunostaining was performed as described^38^. Anti-MMP-9 antibody AF909 was used at 1:30, and anti-goat immunoglobulins coupled to 10-nm gold particles (EMS) were used at 1:100. Sections were counterstained with uranyl acetate and lead citrate and imaged as above.

### Zymography

Frozen thymi were thawed on ice, homogenized, lysed, purified by gelatin-Sepharose affinity chromatography, separated by gel electrophoresis, and analyzed by gelatin zymography as described previously^39^,^40^. As an internal control (IC), a known amount of recombinant human proMMP-9ΔOGHem (MMP-9 deletion mutant lacking hemopexin and O-glycosylation domains) was added to each sample. For the analysis of MMP-9 production in *ex vivo* stimulated cells, stimulations were carried out in serum-free synthetic medium (TexMACS, Miltenyi Biotec) for indicated periods of time, and supernatants were frozen at −80 °C. Thawed samples were purified and analyzed as above. Band densitometry was performed using ImageJ ver. 1.51a modules “Plot Lanes” and “Wand/Tracing Tool” in photographic images of Coomassie Blue-stained zymography gels rotated 0–5° to produce near-vertical bands and cropped to the shown area. No other image manipulations such as brightness or contrast adjustments were performed.

## Results

### Thymic T cell development in the absence of MMP-9

**Figure 1:**
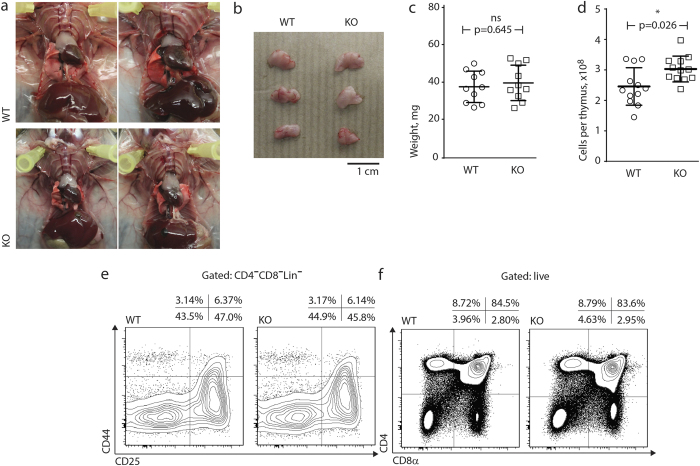
Thymic morphology, cellularity and frequency of major cell subsets. (**a**) Gross anatomy of thymi in wild-type (top) and MMP-9-deficient (bottom) mice. Photographic images show two 4-week old mice per genotype, dissected to expose the thymi. Representative of ten animals per genotype. (**b**) Thymus size and gross morphology in wild-type and MMP-9-deficient mice. Organs from three individual animals per genotype were extracted and photographed. Representative of ten animals per genotype. (**c**) Thymus weight in wild-type and MMP-9-deficient mice. Organs from ten individual animals per genotype were weighed immediately following extraction. p-value results from a two-tailed Mann-Whitney U-test. (**d**) Overall cellularity is slightly increased in MMP-9 deficient mice. Thymi were isolated and pressed through 70 um mesh into PBS. Cell counting was done in three replicates per thymus, and average counts plotted. Data indicative of twelve mice per genotype. p-value results from a two-tailed Mann-Whitney U-test. (**e**) Phenotype of early T cell precursors. Flow cytometry plots represent the distribution of double negative, lineage-negative (CD3, Ly-6G/Ly-6C, CD11b, CD45R, TER-119) thymocytes based on surface CD44 and CD25 staining. Data representative of eight mice per group in two independent experiments. (**f**) Major thymocyte subsets. Flow cytometry plots represent the distribution of all live, single thymocytes based on surface CD4 and CD8 staining. Data representative of twelve mice per group in three independent experiments.

In agreement with published data, MMP-9 deficiency alone did not result in any obvious abnormalities in thymic size and gross morphology (refs. 17,34 and Fig. 1a–c), although there was an approx. 10% increase in thymic cellularity (Fig. 1d).

**Figure 2:**
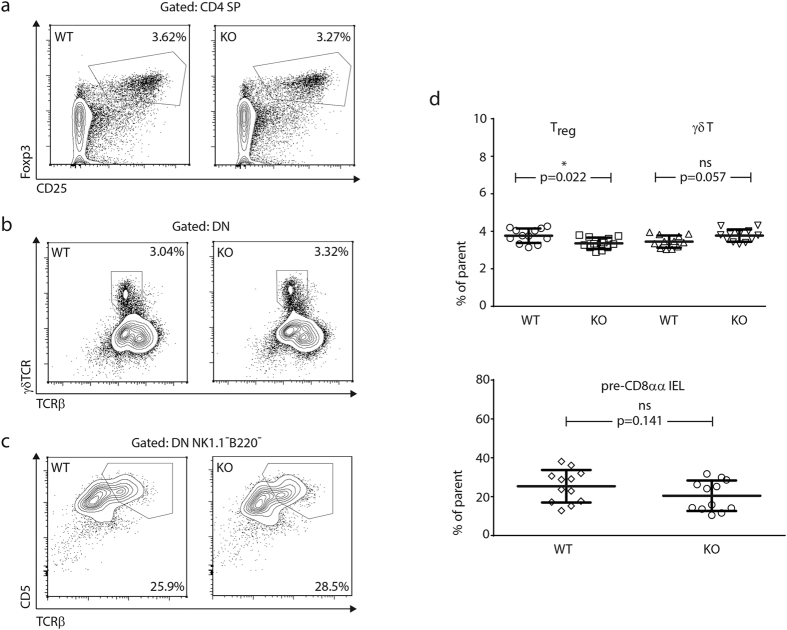
Frequencies of agonist selected thymocytes. (**a**) Thymic precursors of regulatory T cells. Flow cytometry plots represent the distribution of CD4 single-positive thymocytes based on surface CD25 and intracellular Foxp3 staining. Data representative of twelve mice per group in two independent experiments. (**b**) Thymic precursors of γδ T cells. Flow cytometry plots represent the distribution of double negative thymocytes based on surface γδTCR and TCRβ staining. Data representative of twelve mice per group in two independent experiments. (**c**) Thymic precursors of CD8αα intraepithelial lymphocytes. Flow cytometry plots represent the distribution of double-negative, NK1.1- and B220-negative thymocytes based on surface CD5 and TCRβ staining. Data representative of twelve mice per group in two independent experiments. (**d**) Statistical analysis of agonist selected T cell frequencies in the thymus. All significance labels and p-values are derived from two-tailed Mann-Whitney U-tests.

The percentage and composition of immature, CD4/CD8-double negative thymocytes were indistinguishable between MMP-9-sufficient and -deficient mice (Fig. 1e). Proportions of double-positive and single-positive lymphocytes were also identical (Fig. 1f), suggesting an absence of defects in either positive or negative selection. Next, we analyzed the abundance of rare thymocyte populations surviving strong selecting signals and giving rise to agonist-selected subsets. Regulatory T cell precursors were detected within the CD4 single positive population with the use of Foxp3 and CD25 staining (Fig. 2a), and γδ T cell precursors were detected within CD4/CD8-double negative subset by direct staining with a γδTCR antibody (Fig. 2b). The thymocyte subset containing precursors of intraepithelial CD8αα lymphocytes was revealed using TCRαβ and CD5 antibody staining and enumerated within the double negative subset as described^41^ (Fig. 2c). Percentages of precursors of γδ T cells and intraepithelial CD8αα cells were identical, whereas precursors of regulatory T cells displayed a minor (<10%) but reproducible decrease in MMP-9 knockout thymi (Fig. 2d).

**Figure 3:**
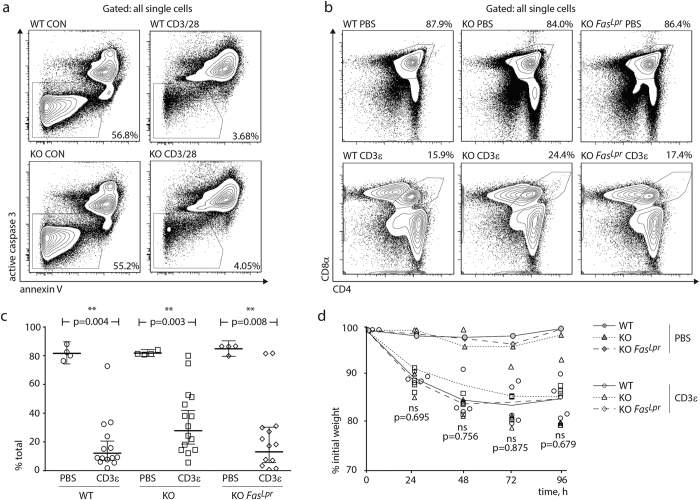
Analysis of negative selection. (**a**) *In vitro* analysis of thymocyte apoptosis. Isolated thymocytes were incubated *in vitro* in complete RPMI in the absence or presence of CD3/CD28-coated magnetic beads, added at 1:1 ratio. Flow cytometry plots represent the distribution of all single thymocytes based on surface annexin V and intracellular active caspase 3 staining. Data representative of four technical replicates per group in four independent experiments (16 wells per group total). (**b**) *In vivo* analysis of thymocyte deletion. Mice were injected with PBS or CD3ε antibody as described in Materials and Methods. Thymi were excised 48 h post injection. Flow cytometry plots represent the distribution of all single thymocytes based on surface CD4 and CD8α staining. Data representative of 13–14 CD3ε-injected mice per group and four PBS injected mice per group in four independent experiments. (**c**) Statistical analysis of double positive thymocyte frequencies in the thymus. All significance labels and p-values are derived from pairwise, two-tailed Mann-Whitney U-tests. (**d**) Body weight loss after CD3ε antibody injection. Four mice per genotype were injected i.p. with CD3ε antibody, and their body weight was recorded over four days in 24-hour intervals. p-values are derived from grouped Kruskal–Wallis one-way analysis of variance between CD3ε-injected WT, MMP9 KO, and *Fas*^*Lpr*^ MMP9 KO mice, at each time point. Gray symbols indicate PBS-injected mice (one per genotype, as in c). Representative of two independent experiments.

In order to test the potential input of MMP-9 into thymocyte apoptosis and clearance during negative selection, we tested the effects of CD3ε antibody treatment *in vivo* and *in vitro*. Incubation of thymocytes with magnetic beads coated with antibodies against CD3 and CD28 resulted in massive apoptosis, as indicated by annexin V-positive and activated caspase 3-positive staining (Fig. 3A). The onset and magnitude of cell death were indistinguishable between MMP-9-sufficient and -deficient samples, indicating that MMP-9 is not required for thymocyte apoptosis in response to strong stimulation. Because MMP-9 function has been associated with macrophage activity, we then analyzed the clearance of apoptotic thymocytes *in vivo*. We injected MMP-9-sufficient and -deficient mice i.p. with anti-CD3ε antibody as described in Materials and Methods, and analyzed thymic deletion after 48 hours. In both cohorts, double-positive thymocytes were effectively cleared, indicating the lack of requirement for MMP-9 activity (Fig. 3b,c). Dulling of surface coreceptors occurred efficiently in both cohorts (data not shown). In light of our previous data indicating a potential synergy between MMP-9 and Fas in the thymus^17^, we also analyzed thymocyte deletion in mice in which the Fas mutation *lpr* was present. In these animals, thymocyte deletion was also unaffected by MMP-9 deficiency (Fig. 3b,c). In all cohorts, CD3ε antibody injection led to a transient weight loss and stress behaviors which are known to be caused by systemic cytokine storm and dehydration. Percentage of weight loss was similar between all genotypes (Fig. 3d).

**Figure 4:**
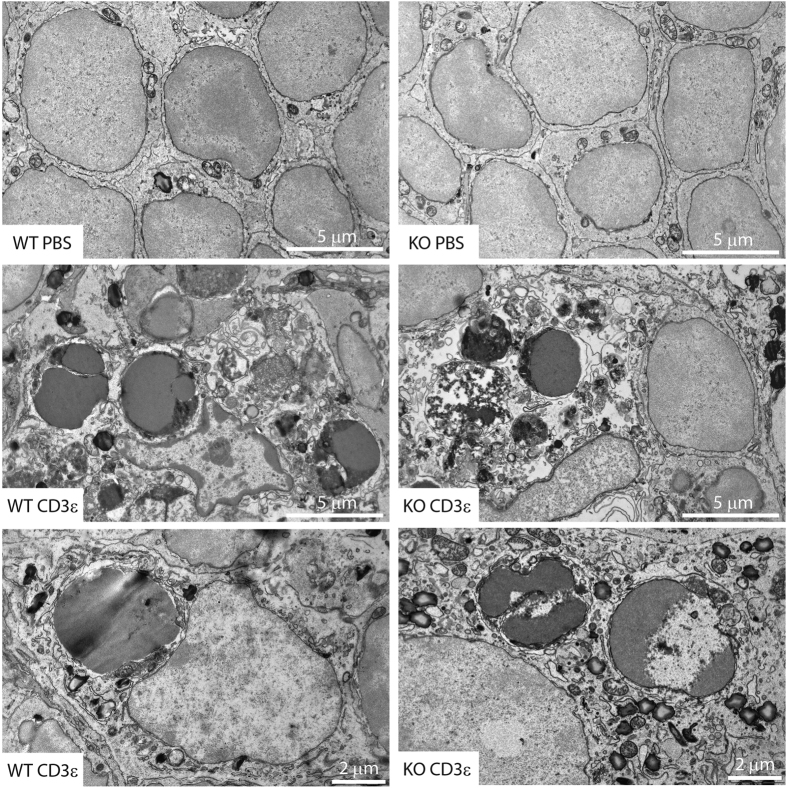
Morphological analysis of thymocyte apoptosis *in situ*. Mice were injected i.p. with PBS (top panel) or CD3ε antibody (middle and bottom panels). Thymi were excised 48 h post injection and processed for electron microscopic analysis as described in Materials and Methods. Images representative of 40–50 individual microscopic images and two individual animals per condition. Bottom panels show engulfment of apoptotic thymocytes. Scale bars, as indicated.

Morphological analysis of thymocyte apoptosis upon *in vivo* application of CD3ε antibody did not reveal any differences between MMP-9-sufficient and -deficient mice. Ultrastructural analysis revealed a massive increase in apoptotic events, as indicated by highly compact, dark, and deformed nuclei, degradation of cell membranes, and autophagy events in the cytoplasm (Fig. 4). Thymocyte phagocytosis events were rare but present in both genotypes (Fig. 4).

### Expression of MMP-9 in the thymus

**Figure 5:**
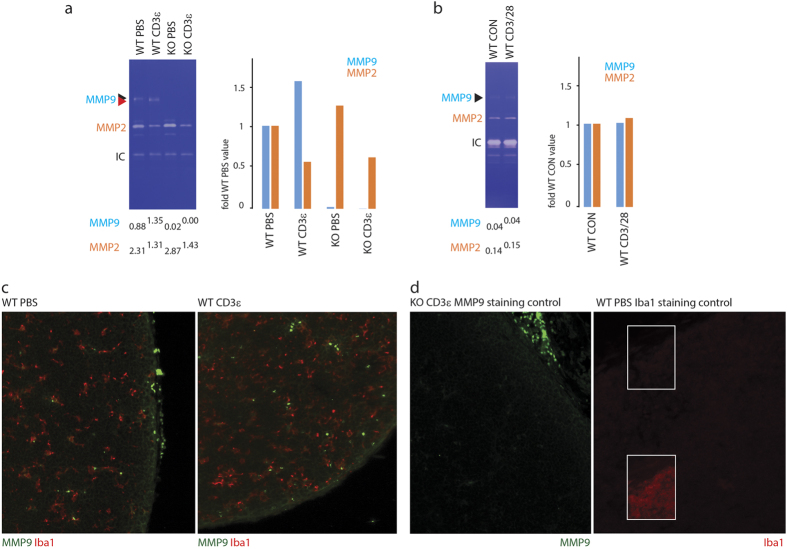
Identification of MMP-9 producing cells in the thymus. (**a**) Mice were injected i.p. with PBS or CD3ε antibody. Thymi were excised 20 h post injection and processed for zymography. Left, zymography data representative of two independent experiments with material from one mouse per condition in each experiment. Locations of MMP-9, MMP-2, and internal control (IC, see Materials and Methods) are indicated. A double arrowhead highlights the band shift characteristic of MMP-9 activation. Densitometry data reflecting band intensities of MMP-9 and MMP-2 relative to their internal controls were calculated using ImageJ (see Materials and Methods), and are indicated below. Right, changes of expression of MMP-9 and MMP-2 relative to wild-type PBS control, derived from densitometry analysis. (**b**) Isolated thymocytes were incubated *in vitro* in serum-free TexMACS medium in the absence or presence of CD3/CD28-coated magnetic beads, added at 1:1 ratio, for 4 h. Culture supernatants were processed for zymography. Data representative of four independent experiments with cells derived from one mouse per experiment. Labeling and densitometry as above. Right, changes of expression of MMP-9 and MMP-2 relative to untreated control sample, derived from densitometry analysis. (**c**) Mice were injected i.p. with PBS (left) or CD3ε antibody (right). Thymi were excised 48 h post injection, fixed in 4% paraformaldehyde, embedded in paraffin, sectioned, and processed for immunohistochemistry. (**d**) Specificity analysis of immunohistochemical staining. MMP-9 specificity control (left): CD3ε-injected sample from an MMP-9-deficient mouse shows non-specific staining of cells attached to the outside of the thymic capsule, and weak diffuse subcapsular staining, and no stained cells in the thymic tissue. Iba1 specificity control (right): PBS-injected sample stained using anti-rabbit secondary antibody without primary antibody shows negligible background staining. The highlighted area is duplicated in the inset after artificial enhancement of brightness and contrast to reveal biological structure.

Because earlier works detected the presence of MMP-9 in thymic tissue, we were interested in identifying cell populations responsible for its expression, particularly among T cell subsets. We confirmed the upregulation and activation of MMP-9 in the thymus following i.p. CD3ε antibody injection (Fig. 5a). It is noteworthy that the upregulation of MMP-9 after stimulation coincided with a reduction of MMP-2 signal, which occurred in both MMP-9-sufficient MMP-9-deficient tissue. A drop in MMP-2 may reflect its active downregulation upon stimulation, or may be indicative of a decrease in the abundance of producing cells. Critically, isolated thymocytes stimulated with CD3/CD28 beads did not produce MMP-9 after 4 h *in vitro* (Fig. 5b). No upregulation was detected after 1, 2, or 18 h *in vitro* stimulation (not shown). Furthermore, MMP-2 signal remained unchanged after *in vitro* stimulation, suggesting that the effect observed in the total thymus (Fig. 5a) is most likely attributable to cells of non-T lineage. Immunohistochemical analysis revealed strong and specific MMP-9 reactivity associated with a small number of round cells enriched in the thymic cortex, and specifically excluded from Iba1-positive cells of dendritic morphology which include cortical and medullar stromal cells and macrophages^42^ (Fig. 5c). In mice injected with CD3ε antibody, MMP-9 displayed a similar distribution pattern although the number of MMP-9-positive cells was increased (Fig. 5c). MMP-9-deficient tissue exhibited non-specific staining of cells attached to the outside of the thymic capsule, and weak diffuse staining of the capsule-proximal cortex, but not of any intrathymic cells (Fig. 5d).

**Figure 6:**
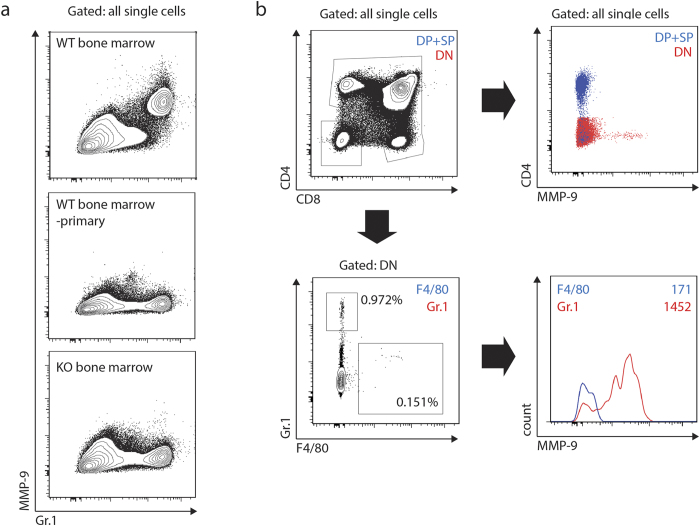
Flow cytometry analysis of MMP-9 expressing populations. (**a**) Specificity of intracellular MMP-9 staining. Plots represent the distribution of all single bone marrow-derived cells based on surface Gr.1 and intracellular MMP-9 staining. Cells were stained for Gr.1, fixed, permeabilized, and stained with MMP-9 antibody and then secondary antibody (top and bottom panels) or secondary antibody alone (middle panel). MMP-9 staining exceeding background level as defined by the signal in the knockout sample was exclusively present in Gr.1-high cells. Data representative of four mice per genotype in two independent experiments. (**b**) Expression of MMP-9 in the thymus. Plots represent the distribution of all single thymocytes based on surface CD4, CD8α, Gr.1, and F4/80 staining, and intracellular MMP-9 staining. Data representative of two mice per experiment and two independent experiments.

**Figure 7:**
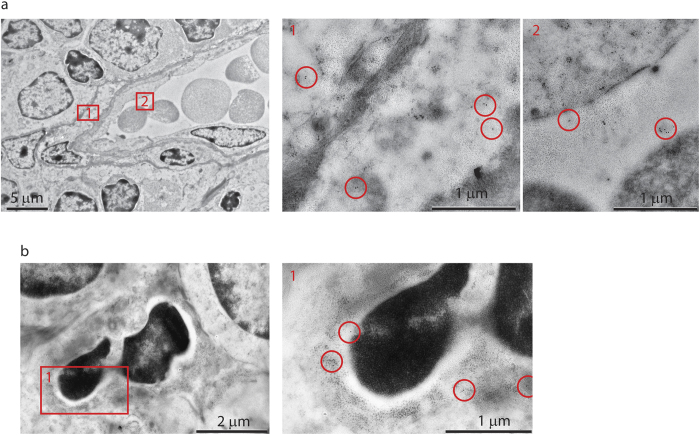
Morphological analysis of MMP-9 expression. Thymi were excised and processed for immunoelectron microscopic analysis as described in Materials and Methods. Numbered areas of interest are enlarged in the higher magnification images on the right, and circles highlight the immunogold label. Images representative of 40–50 individual microscopic images and two individual animals. Scale bars, as indicated.

In order to identify the MMP-9-positive cells, we first validated a commercial polyclonal anti-MMP-9 antibody (see Materials and Methods) for intracellular staining combined with flow cytometry. The antibody strongly stained Gr.1^high^ cells in bone marrow (Fig. 6a) and lymph nodes (data not shown) of wild type mice. In the thymus, the immunoreactivity was exclusively present in a small population of Gr.1^high^, CD4/CD8 double-negative cells corresponding to either thymus-resident neutrophils or neutrophils confined within intrathymic blood vessels^43^ (Fig. 6b), and no immunoreactivity was associated with any T cell precursor subset including single- or double-positive thymocytes. These findings were further confirmed by immunoelectron microscopy which readily detected MMP-9 in perivascular cells, in the lumen of thymic vessels (Fig. 7a), and in tissue-resident polymorphonuclear leukocytes (Fig. 7b) but did not reveal MMP-9 reactivity in apoptotic or non-apoptotic thymocytes from anti-CD3ε injected or non-injected mice.

## Discussion

Development of immature T cells in the thymus involves a series of stimulatory interactions with thymus-resident cells which are necessary for the survival, proliferation, and selection of T cells and ultimately for the formation of a functional T-cell repertoire^44^. During positive selection, interactions with weak self-ligands provide tonic signals required for survival, and the lack of such stimuli, e.g. in immature T cells expressing dysfunctional T cell receptors, results in death by neglect. Conversely, during negative selection interactions with strong self-ligands result in the induction of apoptosis and are required for the elimination of self-reactive clones from the mature T cell repertoire. Specific small subsets of immature T cells can survive strong stimulation by self-antigens in the thymus, and develop into several highly specialized, “agonist-selected” cell types, such as regulatory T cells and several others^45^. Changes in the cellular perception of selecting signals lead to profound developmental abnormalities and can result in immune deficiencies and autoimmunity. Dampening of T cell receptor signaling by the adaptor protein Themis and tyrosine phosphatase Shp-1 has been shown to establish the survival threshold between positive and negative selection, and lack of either of these molecules severely reduced thymic output due to increased signaling in response to weak ligands and increased negative selection^37^,^46^,^47^. Interference with thymocyte apoptosis results in the survival of self-reactive T cell specificities and leads to autoimmune manifestations^48^. Deletion of the transcription factor Aire which is required for the expression of non-thymic antigens by thymic epithelial cells causes a restriction in the repertoire of self-antigens available for negative selection and manifests in a systemic autoimmune syndrome^49^,^50^.

Stimulation of developing thymocytes by systemic application of anti-CD3ε antibody mimics the extreme case of negative selection in TCR-polyclonal mice and results in massive induction of thymocyte death *in vivo* and *in vitro*^51^,^52^. Apoptotic cells in the thymic cortex closely associate with macrophages which are actively recruited to the thymus following stimulation by antibody^53^. Published data indicated an increase in MMP-9 expression in the thymus following CD3ε antibody injection, although specific increase in developing T cells was not reported^34^. Because developing thymocytes are the major subset responding to CD3ε stimulation in the thymus, we hypothesized that these cells may upregulate MMP-9. The biological significance of such upregulation could rely on generation of additional peptides for selection, processing of cell surface receptors and subsequent modification of cellular signaling, regulation of cell contact duration, or other mechanisms.

We have previously reported that the deletion of MMP-9 in mice harboring the *lpr* mutation in the *Fas* gene resulted in an increase in thymus size, although it remains unknown if this effect is due to increased proliferation in the thymus, less efficient selection, or other mechanisms^17^. Fas is expressed in thymocytes^54^, acts in *cis*^55^, and is important for cell clearance in both positive and negative selection^56^,^57^,^58^, although it appears to be involved in the elimination of a relatively small percentage of selected thymocytes and only in response to relatively weak negatively selecting signals^56^,^59^,^60^,^61^. A separate Fas-independent, Bim-dependent mechanism has been shown to be activated in conjunction with negative selection^62^,^63^,^64^. Intriguingly, at least one matrix metalloproteinase, MMP-7/matrilysin cleaves both Fas and FasL^65^,^66^. MMP-7-mediated cleavage of Fas reduces susceptibility of target cells to apoptosis induced by cytotoxic T cells, Fas antibody, and chemical stimuli^65^,^66^,^67^, whereas cleavage of membrane-associated FasL produces a soluble fragment which is biologically active in inducing target cell apoptosis^68^. Enhancement of thymus hyperplasia in Fas^*Lpr*^ mice by MMP-9 deficiency hinted to the possibility of involvement of the latter into thymocyte clearance.

We have extensively tested both hypotheses by comparing thymocyte development in mice sufficient and deficient for MMP-9. Our data clearly suggest that thymocyte development does not require MMP-9 activity. All thymic subsets were represented at appropriate proportions in MMP-9-deficient mice, with an exception of a <10% decrease in regulatory T cell percentage, and *in vitro* stimulation of thymocytes from both mouse cohorts revealed no differences in developmental outcomes. Perhaps more surprisingly, MMP-9 deficiency did not result in any abnormalities in the clearance of apoptotic thymocytes in mice both in C57BL/6 mice and Fas^*Lpr*^ mice. A small increase in thymic cellularity and a small decrease in thymic regulatory T cell percentage suggest that an MMP-9-dependent mechanism may play an accessory role in the agonist selection of this particular subset and in thymocyte proliferation. One candidate molecule is TGFβ which can be released from its latent precursor form by MMP-9-mediated cleavage^12^. However, while TGFβ is central to the peripheral conversion and maintenance of regulatory T cells, T cell-specific deletion of *Tgfbr2* gene revealed no changes in the thymic development of this subset or in fact any αβ T cell subset, or indeed in thymic cellularity^69^. It is also important to notice that the small increase in thymic cellularity in MMP-9 knockout mice does not translate into an increase in cellularity in peripheral lymphoid organs^17^.

We further concluded that developmental signaling in the thymus does not result in detectable T cell-intrinsic upregulation of MMP-9 expression. While MMP-9 was readily detected in thymic neutrophils, no T cell subset revealed MMP-9 reactivity in either flow cytometry or immunohistochemistry assays. *In vitro* stimulation of isolated thymocytes with CD3/CD28-coated microbeads did not result in upregulation of MMP-9 levels, as demonstrated in zymography experiments.

MMP-9 expression in thymic tissue and its upregulation upon CD3ε antibody injection has been described before, and correlated with neovascularization of the thymus^34^. The authors of that study hypothesized that this mechanism may increase the abundance of macrophages required for the clearance of apoptotic thymocytes. While this may indeed be the case, the functional consequences of MMP-9 dependent neovascularization and additional macrophage influx appear negligible as the lack thereof does not lead to any differences in thymocyte elimination. While more research is needed to better understand the importance of MMP-9 for the biology of thymic macrophages and neutrophils, our data suggest that its role is unrelated to thymocyte development and differentiation. The increased abundance of MMP-9-positive cells after stimulation *in vivo* but not *in vitro* suggests an influx of MMP-9-expressing cells from the bloodstream rather than induction of expression in a thymus-resident population.

Multiple matrix metalloproteinases have been implicated in thymocyte development and homeostasis. Similarly to MMP-9, stromelysins MMP-3, -10, and -11 are induced in thymi of mice injected with CD3ε antibody, and at least one of these, MMP-11 was detected in macrophages associated with apoptotic thymocytes^70^. Membrane-associated MMP-14 is upregulated in human thymocytes incubated on laminin-5 and implicated in the generation of soluble fragment of CD44 thought to induce migration^71^. MMP-19 has been shown to promote thymocyte proliferation specifically in young mice, where its deletion led to a partial block in DP-SP transition^72^. Activity of thymic plasmin which is known to process multiple matrix metalloproteinases including the aforementioned MMP-3^73^, MMP-11^74^, and MMP-14^75^, as well as MMP-9^76^, is increased upon CD3ε antibody injection^34^. A concerted action of MMP-9 and other matrix metalloproteinase(s) such as MMP-11, -14, -19, or other may result in functional redundancy and mask a phenotype of a single MMP knockout. As a final remark, care should be taken when interpreting thymus-specific effects of CD3ε antibody injections on non-T cell subsets which are also highly represented in the bloodstream and peripheral lymphoid organs. Massive cytokine storm caused by antibody-mediated T cell activation^77^ is very likely to strongly affect thymic subpopulations of non-T cells and non-thymus resident T cells, e.g. those located within thymic blood vessels.

## Additional Information

**How to cite this article**: Gounko, N. V. *et al*. Thymocyte development in the absence of matrix metalloproteinase-9/gelatinase B. *Sci. Rep.* **6**, 29852; doi: 10.1038/srep29852 (2016).
